# The feasibility of implementing recovery, psychosocial and pharmacological interventions for psychosis: comparison study

**DOI:** 10.1186/s13012-015-0262-9

**Published:** 2015-05-23

**Authors:** Lian van der Krieke, Victoria Bird, Mary Leamy, Faye Bacon, Rebecca Dunn, Francesca Pesola, Monika Janosik, Clair Le Boutillier, Julie Williams, Mike Slade

**Affiliations:** University Medical Center Groningen, University Center for Psychiatry, University of Groningen, Groningen, The Netherlands; Health Service and Population Research Department, Institute of Psychiatry, Psychology & Neuroscience, King’s College London, London, UK

**Keywords:** Implementation science, Psychosis, Schizophrenia, Clinical guidelines, Interventions

## Abstract

**Background:**

Clinical guidelines for the treatment of people experiencing psychosis have existed for over a decade, but implementation of recommended interventions is limited. Identifying influences on implementation may help to reduce this translational gap. The Structured Assessment of Feasibility (SAFE) measure is a standardised assessment of implementation blocks and enablers. The aim of this study was to characterise and compare the implementation blocks and enablers for recommended psychosis interventions.

**Methods:**

SAFE was used to evaluate and compare three groups of interventions recommended in the 2014 NICE psychosis guideline: pharmacological (43 trials testing 5 interventions), psychosocial (65 trials testing 5 interventions), and recovery (19 trials testing 5 interventions). The 127 trial reports rated with SAFE were supplemented by published intervention manuals, research protocols, trial registrations and design papers. Differences in the number of blocks and enablers across the three interventions were tested statistically, and feasibility profiles were generated.

**Results:**

There was no difference between psychosocial and recovery interventions in the number of blocks or enablers to implementation. Pharmacological interventions (a) had fewer blocks than both psychosocial interventions (*χ*^2^(3) = 133.77, *p* < 0.001) and recovery interventions (*χ*^2^(3) = 104.67, *p* < 0.001) and (b) did not differ in number of enablers from recovery interventions (*χ*^2^(3) = 0.74, *p* = 0.863) but had fewer enablers than psychosocial interventions (*χ*^2^(3) = 28.92, *p* < 0.001). Potential adverse events associated with the intervention tend to be a block for pharmacological interventions, whereas complexity of the intervention was the most consistent block for recovery and psychosocial interventions.

**Conclusions:**

Feasibility profiles show that pharmacological interventions are relatively easy to implement but can sometimes involve risks. Psychosocial and recovery interventions are relatively complex but tend to be more flexible and more often manualised. SAFE ratings can contribute to tackling the current implementation challenges in mental health services, by providing a reporting guideline structure for researchers to maximise the potential for implementation and by informing prioritisation decisions by clinical guideline developers and service managers.

## Background

Clinical guidelines for the treatment of people experiencing psychosis in the UK were published by the National Institute for Health and Care Excellence (NICE) in 2014 [1], updating earlier guidelines published in 2002 and 2009. The guidelines cover a range of evidence-based pharmacological and psychosocial interventions. However, the implementation of these interventions, particularly the psychosocial interventions, is limited [2–4]. A 2007 report of the Healthcare Commission and the Commission for Social Care Inspection stated that only 53 % of schizophrenia patients living in Britain received NICE-recommended family intervention, and only 46 % had access to NICE-recommended cognitive behavioural therapy [5]. A more recent survey study among 187 schizophrenia patients in North-West England showed that only 7 % of patients were offered and 5 % received cognitive behavioural therapy. The percentages for family intervention were again lower: 3 % of patients were offered family intervention and only 1 % received it [6].

Guideline development and policy-making focus on systematic reviews of efficacy and cost-effectiveness, with the feasibility of an intervention somewhat ignored, or considered following the recommendations [7]. This relative disregard of the feasibility of the intervention seems unhelpful, given the importance of feasibility in implementation theory. The Consolidated Framework For Implementation Research (CFIR), which provides a comprehensive theoretical framework of key implementation constructs, comprises five major interacting domains that influence implementation effectiveness [8]: outer setting (the economic, political and social context within which an organisation resides), inner setting (features of structural, political and cultural contexts), characteristics of individuals involved (who are affected by cultural, organisational, professional and individual mindsets, norms, interests and affiliations), the process of implementation (implementation may be actively promoted with support from the inner or outer setting, there may be sub-processes planned or unplanned, with a linear or non-linear course, etc.) and intervention characteristics. An intervention can be more or less feasible depending on, for instance, the complexity of the intervention or its adaptability to a particular setting. Since feasibility of the intervention is identified as a domain that influence implementation processes, it needs to be taken into account when decisions are made about prioritisation or recommendation of interventions. Therefore, the implementation feasibility of interventions is the focus of the current study.

Our aim was to evaluate and compare the feasibility of different interventions included within the NICE psychosis guidelines, using a standardised measure of feasibility called Structured Assessment of Feasibility (SAFE; [7]). We compared the feasibility of pharmacological, psychosocial and recovery interventions.

## Methods

### Measures

The SAFE measure is a 16-item assessment of the feasibility of an intervention for routine implementation [7]. In this measure, feasibility is defined as the cumulative impact of different influences that affect the implementation of an intervention within a specific health care practice. Each SAFE item was identified from implementation research, classified as either a block (eight items) or an enabler (eight items) of implementation and rated on a four-point scale (Yes, Partial, No, Unable to rate).

The eight-block items are:

- B1—Training: Does staff require specific training to deliver the intervention?
- B2—Complexity: Is the intervention complex?
- B3—Time: Is the intervention time-consuming to provide?
- B4—Support: Does the intervention include/require ongoing support and supervision
- B5—Personnel: Does the intervention require additional human resources?
- B6—Material: Does the intervention require additional material resources?
- B7—Costs: Is the intervention costly?
- B8—Harms: Are there any known serious or adverse events associated with the intervention?

The eight enabler items are:

- E1—Population: Is the intervention applicable to the population of interest?
- E2—Manualisation: Is the intervention manualised?
- E3—Flexibility: Is the intervention flexible?
- E4—Effectiveness: Is the intervention likely to be effective?
- E5—Saving: Is the intervention cost-saving?
- E6—Goals: Do the intended goals of the intervention match the prioritised goals of the NHS?
- E7—Pilot: Can the intervention be piloted?
- E8—Reversibility: Is the intervention reversible?

SAFE has ‘excellent’ [9] inter-rater reliability (kappa = 0.84, 95 % CI 0.79–0.89) and test-retest reliability (kappa = 0.89, 95 % CI 0.85–0.93) [7].

### Procedure

We reviewed the 2014 NICE guidelines for psychosis and schizophrenia in adults [1] to identify recommended interventions. Interventions were categorised into pharmacological, psychosocial and recovery interventions. This categorisation was guided by the aim of distinguishing between (1) intervention type, namely medication versus non-medication-based therapy, and (2) targeted outcomes, namely clinical recovery (e.g. symptomatic relief, reduced hospitalisation) versus personal recovery (i.e. being able to live a meaningful and satisfying life beyond the illness [10]). Pharmacological interventions were defined as medication based and focused on clinical recovery outcome, psychosocial interventions as non-medication based and focused on clinical recovery outcomes and recovery interventions as non-medication based and focused on personal recovery outcomes.

Five interventions from each intervention category which had the strongest recommendation in the NICE guidelines, as reflected in the wording of the recommendation (e.g. ‘offer’, ‘should be offered’ or ‘consider offering’ versus ‘do not routinely offer’ or ‘do not offer’) were selected for review. For each of these 15 interventions, we searched all full reports of randomised controlled trials (RCTs), published from 2000 onwards that are referred to in the NICE guidelines. In the case of multiple parallel or follow-up publications of the same study, we selected the first publication. Trial reports were supplemented by published intervention manuals, research protocols, trial registrations and design papers referred to in the trial reports. Trials were independently rated using SAFE by two raters (RD and LvdK), who double-rated 10 % of trials to check concordance.

### Analysis

Chi-square tests were used to compare the overall proportion of implementation blocks and enablers across intervention categories. Although the SAFE manual recommends to use individual item scores instead of summary scores to account for unequal weight of items, in this first analysis, we did use the total number of blocks and enablers in order to gain a global overview of blocks and enablers across the rather broad intervention categories. In the chi-square tests, interventions (pharmacological, psychosocial and recovery) were crossed with rating categories (Yes, Partial, No, Unable to rate), for both blocks and enablers. So, we compared the weighted frequency of Yes (*n* = 10), Partial (*n* = 45), No (*n* = 281) and Unable (*n* = 8) ratings for blocks on pharmacological interventions to the weighted frequency of Yes (*n* = 77), Partial (*n* = 110), No (*n* = 231) and Unable (*n* = 102) ratings for blocks on psychosocial interventions versus the weighted frequency of Yes (*n* = 38), Partial (*n* = 30), No (*n* = 63) and Unable (*n* = 21) ratings for blocks on recovery interventions. In addition, we compared the weighted frequency of Yes (*n* = 210), Partial (*n* = 38), No (*n* = 51) and Unable (*n* = 45) ratings for enablers on pharmacological interventions to the weighted frequency of Yes (*n* = 358), Partial (*n* = 69), No (*n* = 23) and Unable (*n* = 70) ratings for enablers on psychosocial interventions versus the weighted frequency of Yes (*n* = 108), Partial (*n* = 17), No (*n* = 8) and Unable (*n* = 19) ratings for enablers on recovery interventions. Post hoc tests were used to examine the proportion of blocks and enablers in pairs: pharmacological interventions versus psychosocial interventions, psychosocial interventions versus recovery interventions and recovery interventions versus pharmacological interventions. For the post hoc tests, a Bonferroni-corrected significance level of 0.01 was taken.

Fisher’s exact tests were used to make pairwise comparisons of blocks and enablers on item level (i.e. separate analyses for B1 to B8 and E1 to E8). In the latter comparisons, the four response categories (Yes, Partial, No, Unable to rate) were restricted to the two categories Yes versus ‘non-Yes’ because of small cell counts. So, we crossed ‘Yes’ versus ‘non-Yes’ with intervention category (pharmacological, psychosocial, recovery), for each block and each enabler separately. For the pairwise comparisons, the significance level was corrected to *p* < 0.002. As a measure of effect size, we calculated Mantel-Haenszel odds ratios, which provide a pooled odds ratio across strata [11].

Graphic profiles of implementation blocks and enablers were created to visualise the proportion of reported blocks and enablers for each SAFE item per intervention category. All four response categories are presented (Yes, Partly, No, Unable to rate). The graphic profiles were modified from the Cochrane ‘risk of bias’ graph recommended for use in systematic reviews, which shows the proportion of low, high and unclear risk of bias in the design of a study [12].

## Results

The 15 included interventions and 127 related RCTs are shown in Table 1.

**Table 1:** Included interventions for psychosis (n = 15)

Recovery interventions	RCTs	Psychosocial interventions	RCTs	Pharmacological interventions	RCTs
Illness Management and Recovery (IMR)	4	Cognitive behavioural therapy (CBT)	25	Acute treatment—oral antipsychotics not otherwise specified	24
Wellness Recovery Action Planning (WRAP)	1	Family intervention (FI)	20	Relapse prevention—oral antipsychotics not otherwise specified	8
Individual Placement and Support (IPS)	12	Behavioural lifestyle intervention (combined physical activity and healthy eating)	15	Relapse prevention—depot medication	2
Recovery Workbook	1	Arts therapy	4	Treatment resistance—clozapine	2
Building Recovery of Individual Dreams and Goals through Education and Support (BRIDGES)	1	Psychoanalytic/psychodynamic therapy	1	Smoking cessation—bupropion or varenicline	7
Total RCTs:	19		65		43

Concordance on SAFE rating was kappa = 0.89 (95 % CI 0.83–0.94), which can be considered ‘excellent’ [9].

**Fig. 1:**
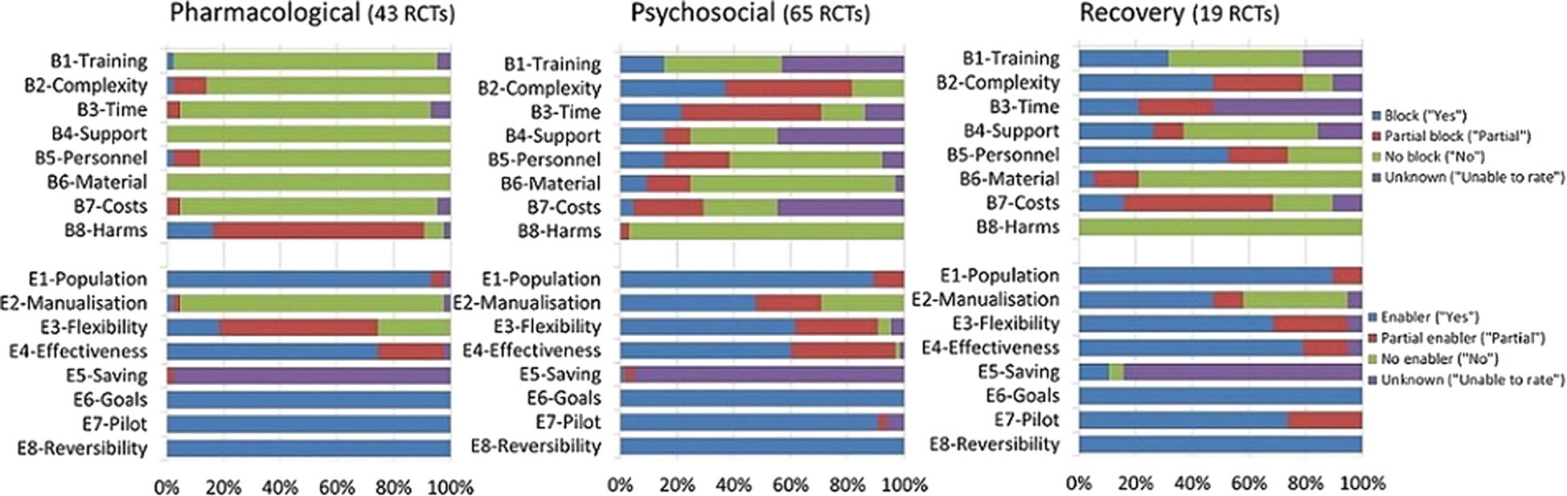
Feasibility profiles showing pooled blocks and enablers for three categories of intervention

Visual profiles of proportion of implementation blocks and enablers for each intervention category are shown in Fig. 1. These profiles show that psychosocial interventions and recovery interventions have a relatively broad range of blocks and enablers that partly overlap, whereas pharmacological interventions have a less varied profile.

There was a significant difference in total number of blocks (*χ*^2^(6) = 161.05, *p* < 0.000) and enablers (*χ*^2^(6) = 33.02, *p* < 0.000) between the pharmacological, psychosocial and recovery intervention categories. Post hoc tests showed no difference between psychosocial interventions and recovery interventions for blocks (*χ*^2^(3) = 9.65, *p* = 0.02) or enablers (*χ*^2^(3) = 0.74, *p* = 0.863). Pharmacological interventions had fewer blocks than both psychosocial interventions (*χ*^2^(3) = 133.77, *p* < 0.001) and recovery interventions (χ(3)^2^ = 104.67, *p* < 0.001). Pharmacological interventions did not differ in number of enablers from recovery interventions (*χ*^2^ = 0.74, *p* = 0.863) but had fewer enablers than psychosocial interventions (*χ*^2^(3) = 28.92, *p* < 0.001).

Differences between specific blocks and enablers across intervention categories are shown in Table 2, showing for example that pharmacological interventions had fewer blocks relating to complexity than psychosocial interventions.

**Table 2:** Pairwise comparisons of blocks and enablers between intervention categories

Item	Pharmacological versus psychosocial	Psychosocial versus recovery	Pharmacological versus recovery
	*χ* ^2^(df), *p*, </>, odds ratio	*χ* ^2^(df), *p*, </>, odds ratio	*χ* ^2^(df), *p*, </>, odds ratio
B1—Training	4.825(1)	2.501(1)	11.259(1)
	0.047	0.180	0.002
B2—Complexity	17.413(1)	0.673(1)	19.763(1)
	<0.001	0.435	<0.001
	<	-	<
	24.585	-	37.800
B3—Time	-	0.002(1)	-
	N/A^a^	1.000	N/A^a^
B4—Support	-	1.198(1)	-
	N/A^a^	0.313	N/A^a^
B5—Personnel	4.825(1)	11.244(1)	22.850(1)
	0.047	0.002	<0.001
	-	-	<
	-	-	46.667
B6—Material	-	0.303(1)	-
	N/A^a^	1.000	N/A^a^
	-	0.546	-
B7—Costs	-	2.768(1)	-
	N/A^a^	0.126	N/A^a^
B8—Harms	N/A^a^	N/A^a^	N/A^a^
E1- Population	0.443(1)	0.001(1)	0.224(1)
	0.737	1.000	0.638
E2- Manualisation	25.546(1)	0.001(1)	19.763(1)
	<0.001	1.000	<0.001
	<	-	<
	38.294	-	37.800
E3- Flexibility	19.320(1)	0.299(1)	14.600(1)
	<0.001	0.788	<0.001
	<	-	<
	7.000	-	9.479
E4- Effectiveness	2.389(1)	2.299(1)	0.147(1)
	0.149	0.176	1.000
E5—Saving	-	3.449(1)	-
	N/A^a^	0.127	N/A^a^
E6—Goals	N/A^a^	N/A^a^	N/A^a^
E7—Pilot	-	3.771(1)	-
	N/A^a^	0.114	N/A^a^
E8—Reversibility	N/A^a^	N/A^a^	N/A^a^

^a^Analyses could not be run because at least one of the frequencies was zero

## Discussion

This study investigated the implementation feasibility of pharmacological, psychosocial and recovery interventions recommended in the NICE guidelines for adults experiencing psychosis. Psychosocial and recovery interventions have the same number of blocks and enablers, and they differ in their profile from pharmacological interventions. Both psychosocial interventions and recovery interventions can be complex and consist of multiple interacting components. In addition, recovery interventions can require additional staff to be provided. However, recovery and psychosocial interventions have the advantage that they are often manualised and that they can be easily tailored to a specific situation or context. Pharmacological interventions have fewer blocks, but visual profiles show that they tend to have a higher risk for adverse events.

### Strengths and limitations

This is the first study to assess the implementation feasibility of interventions recommended in the NICE guidelines for people with psychosis, thus offering an approach which can inform both guideline development processes and routine implementation of interventions. We identify five limitations. First, not all recently published studies were included. The evidence for psychosocial and recovery interventions was updated up to the publication date of the NICE guidelines, which was February 2014. This is one reason for the small number of RCT reports of recovery interventions reviewed. Moreover, in contrast to the psychosocial and recovery interventions, the evidence on pharmacological interventions was not updated in the 2014 NICE guidelines. Therefore, for this intervention category, we only included publications up to 2009, which might have introduced a bias to our sample of pharmacological papers.

Second, our analysis of implementation feasibility of interventions was based on published documents, so SAFE ratings may be influenced by reporting quality rather than the actual intervention. We addressed this issue by accessing published manuals, design papers, research protocols and trial registrations alongside trial reports. However, in some cases, these additional documents did not provide all information needed. For instance, information about costs and cost savings was often limited. In combination with the relatively high threshold used in SAFE ratings for an intervention to be identified as costly, this may partly explain why no differences in cost and cost savings were found between intervention categories. Some specific items, such as costs, may therefore benefit from more detailed analysis to inform recommendations. Related to this limitation, reports of RCTs describe what happened in the context of a trial, when implementation might be more standardised and less flexible than in routine clinical practice. For example, in most reviewed pharmacological trials, medication was prescribed either in a fixed dosage (i.e. the same for each service user) or in a combination of partly fixed and partly variable dosage (e.g. a fixed dosage for the first few weeks, and a variable dosage in later stages of the trial), whereas changes to dosage outside of a trial context may be more variable. Our study might therefore have underestimated the actual flexibility of interventions. However, our approach to using RCTs is consistent with the methodology used to develop guidelines, in that the efficacy estimates come from controlled conditions, which may differ from the efficacy within routine implementation.

A third limitation concerns rating the extent to which interventions were manualised. The difference in manualisation between psychosocial and recovery interventions versus pharmacological interventions may be explained by the fact that in almost all pharmacological interventions, the only structured part was the specified medication dosages. Although dosage may be a crucial component in the delivery of a pharmacological intervention, NICE guidelines emphasise that pharmacotherapy is a trajectory including discussion of risks and benefits and careful recording and monitoring of response and side effects. If this trajectory was not structured or specified in most trial reports, we considered the pharmacological intervention not to be manualised. The implication of our approach may be an underestimation of manualisation in pharmacological interventions.

The fourth limitation is that we could not perform quantitative analyses for all comparisons of blocks and enablers because of empty cells (i.e. zero counts for blocks and enablers) for some items. In these cases, we had to rely on the visual profiles shown in Fig. 1. A final limitation is that we rated only the 15 interventions listed in the NICE guidelines with the strongest evidence so did not provide a comprehensive overview of all interventions.

### Implications

This study offers an approach which may contribute to tackling the current implementation challenges in psychiatry by addressing one of the major implementation domains identified in implementation theory, namely feasibility of the intervention [8]. Assessment of intervention feasibility can inform the development process of clinical guidelines. Current guidelines are primarily based on evidence reviews with a focus on efficacy and cost-effectiveness [13]. We propose that guideline development would benefit from more weight being put on the feasibility of interventions. Incorporating SAFE ratings into the evidence appraisal would provide a metric for guideline developers to take into account those aspects that might hamper or facilitate successful implementation. For instance, if an intervention A has higher efficacy than intervention B but also has a higher risk for doing harm, then this may be a reason to give higher recommendation to intervention B. In addition, if an intervention is cost-effective but has also found to be highly complex in terms of implementation feasibility, this should be balanced in the recommendations. Balanced recommendations can be made which differentiate between settings in which implementation is more or less feasible.

SAFE items were sometimes scored as ‘unable to rate’ because trial reports and manuals did not provide complete information. Information was particularly lacking regarding potential blocks in staff training, time spent on the delivery of the intervention, ongoing supervision and cost-saving potential of the intervention. Future studies might benefit from using SAFE reporting guidelines, which parallel the SAFE assessment items [7], in a way that the PRISMA [14] and CONSORT [15] statements guide the reporting of systematic reviews and RCTs. These reporting guidelines may help researchers to put more emphasis on feasibility issues, both in the initial design and the final reporting of trials.

Finally, feasibility profiles have relevance for service development as they can support service managers in identifying the important and evidence-based feasibility issues that are likely to arise during translation of evidence into practice. SAFE ratings can provide a pre-implementation assessment; an important step in the implementation process of evidence-based interventions into everyday clinical practice [16–18]. For instance, looking at the feasibility profiles of recovery interventions in Fig. 1, service managers can conclude that, for the included interventions, (1) they can be sure that recovery interventions are applicable to service users with psychosis, that the goals of the intervention match the prioritised goals of the NHS, that there is evidence of effectiveness (these items all have long blue bars indicating that they are rated as enablers), (2) they do not have to worry about negative consequences when the interventions would be stopped (low risk of irreversibility), (3) the implementation of these interventions can benefit from the fact that recovery interventions are often manualised and can be tailored to context and situation, but that (4) they should take into account that additional staff is needed to provide the interventions and that the interventions often consist of multiple interacting components, which might mean that they have to appoint a skilled person to supervise implementation processes. Assessment of potential blocks and enablers, such as staff time to tailor interventions or to manage the interaction between components of a complex intervention, or the availability of manuals, allows for the creation of an implementation plan in which priorities are set and problems can be anticipated. This enables service managers to allocate resources efficiently and to speed the uptake of interventions by clinical staff.

## Conclusion

SAFE ratings have the potential to be used during the clinical guideline development process to make feasibility assessment a routine component of evidence-based appraisal. The study highlighted how more attention should focus on detailing the complexity and potential harms of an intervention when reporting the results of a study. Service managers can use SAFE profiles to inform decisions about resource allocation so that more patients have access to recommended treatment.

## Abbreviations

NICE: National Institute for Health and Care Excellence
SAFE: Structured Assessment of Feasibility
RCT: randomised controlled trial

## References

[1] National Institute for Health and Care Excellence (2014). Psychosis and Schizophrenia in adults. The NICE guideline on treatment and management. CG178.

[2] Sederer LI (2009). Science to practice: making what we know what we actually do. Schizophr Bull.

[3] Barbui C, Girlanda F, Ay E, Cipriani A, Becker T, Koesters M (2014). Implementation of treatment guidelines for specialist mental health care. Cochrane Database Syst Rev..

[4] Michie S, Pilling S, Garety P, Whitty P, Eccles MP, Johnston M (2007). Difficulties implementing a mental health guideline: an exploratory investigation using psychological theory. Implement Sci..

[5] Healthcare Commission and the Commission for Social Care Inspection (2009). No voice, no choice: a joint review of adult community mental health services in England.

[6] Haddock G, Eisner E, Boone C, Davies G, Coogan C, Barrowclough C (2014). An investigation of the implementation of NICE-recommended CBT interventions for people with schizophrenia. J Ment Health.

[7] Bird VJ, Le Boutillier C, Leamy M, Williams J, Bradstreet S, Slade M (2014). Evaluating the feasibility of complex interventions in mental health services: standardised measure and reporting guidelines. Br J Psychiatry..

[8] Damschroder LJ, Aron DC, Keith RE, Kirsh SR, Alexander JA, Lowery JC. Fostering implementation of health services research findings into practice: a consolidated framework for advancing implementation science. Implement Sci. 2009;4:50-5908-4-50. 10.1186/1748-5908-4-50 PMC2736161 19664226

[9] Landis JR, Koch GG (1977). The measurement of observer agreement for categorical data. Biometrics.

[10] Slade M (2009). Personal recovery and mental illness. A guide for mental health professionals.

[11] Mannocci A (2009). The Mantel-Haenszel procedure. 50 years of the statistical method for confounders control. Ital J. Public Health.

[12] Higgins JPT, Green S. Cochrane Handbook for Systematic Reviews of Interventions Version 5.1.0 [updated March 2011]. 2011.

[13] Berry K, Haddock G (2008). The implementation of the NICE guidelines for schizophrenia: barriers to the implementation of psychological interventions and recommendations for the future. Psychol Psychother.

[14] Moher D, Liberati A, Tetzlaff J, Altman DG, PRISMA Group (2009). Preferred reporting items for systematic reviews and meta-analyses: the PRISMA statement. J Clin Epidemiol..

[15] Schulz KF, Altman DG, Moher D, CONSORT Group (2010). CONSORT 2010 statement: updated guidelines for reporting parallel group randomised trials. PLoS Med.

[16] Stetler CB, Legro MW, Wallace CM, Bowman C, Guihan M, Hagedorn H (2006). The role of formative evaluation in implementation research and the QUERI experience. J Gen Intern Med..

[17] Mendel P, Meredith LS, Schoenbaum M, Sherbourne CD, Wells KB (2008). Interventions in organizational and community context: a framework for building evidence on dissemination and implementation in health services research. Adm Policy Ment Health.

[18] Kilbourne AM, Neumann MS, Pincus HA, Bauer MS, Stall R (2007). Implementing evidence-based interventions in health care: application of the replicating effective programs framework. Implement Sci..

